# 429. Risk of COVID-19 Related Deaths for SARS-CoV-2 Omicron (B.1.1.529) Compared With Delta (B.1.617.2): A Retrospective Cohort Study

**DOI:** 10.1093/ofid/ofad500.499

**Published:** 2023-11-27

**Authors:** Shon Paulson, Jomel Raju

**Affiliations:** St. Joseph's College of Pharmacy, Thrissur, Kerala, India; St. Joseph's College of Pharmacy, Cherthala, Pala, Kerala, India

## Abstract

**Background:**

The study aimed to assess the risk of covid-19 death after infection with omicron BA.1 compared with the delta (B.1.617.2) variant of the SARS-CoV-2 virus in India.

**Methods:**

The retrospective cohort study is conducted in the state of Kerala, India by retrieving data from the COVID-19 database of the Directorate of Health Services (DHS), Government of Kerala. The main outcome measure was covid-19 death as identified from death certification records. The exposure of interest was the SARS-CoV-2 variant identified PCR positive tests taken in the community sampling and analyzed by the state-approved laboratories. Cause-specific Cox proportional hazard regression models (censoring non-covid-19 deaths) were adjusted for sex, age, vaccination status, previous infection, calendar time, ethnicity, and comorbidities. Interactions between variants and sex, age, vaccination status, and comorbidities were also investigated.

**Results:**

A total of 1035143 people aged 18-100 years who tested positive for SARS-CoV-2 under the surveillance program for COVID-19 and had an infection identified as omicron BA.1 or delta variants of SARS-CoV-2 were included in the study. The risk of covid-19 death was 66% lower (95% confidence interval 54% to 75%) for omicron BA.1 compared with delta after adjusting for a wide range of potential confounders. The reduction in the risk of covid-19 death for omicron compared with delta was more pronounced in people aged 18-59 years (number of deaths: delta=46, omicron=11; hazard ratio 0.14, 95% confidence interval 0.07 to 0.27) than in those aged ≥70 years (number of deaths: delta=113, omicron=135; hazard ratio 0.44, 95% confidence interval 0.32 to 0.61, P< 0.0001). No evidence of a difference in risk was found between the variant and the number of comorbidities.

**Conclusion:**

The results support earlier studies showing a reduction in the severity of infection with omicron BA.1 compared with a delta in terms of hospital admission. This study extends the research to also show a reduction in the risk of covid-19 death for the omicron variant compared with the delta variant.

**Disclosures:**

**All Authors**: No reported disclosures

